# The Dunkin Hartley Guinea Pig Is a Model of Primary Osteoarthritis That Also Exhibits Early Onset Myofiber Remodeling That Resembles Human Musculoskeletal Aging

**DOI:** 10.3389/fphys.2020.571372

**Published:** 2020-10-29

**Authors:** Robert V. Musci, Maureen A. Walsh, Adam R. Konopka, Christopher A. Wolff, Frederick F. Peelor, Raoul F. Reiser, Kelly S. Santangelo, Karyn L. Hamilton

**Affiliations:** ^1^Department of Health and Exercise Science, Colorado State University, Fort Collins, CO, United States; ^2^Department of Medicine, University of Wisconsin-Madison, Madison, WI, United States; ^3^GRECC, William S Middleton Memorial Veterans Hospital, Madison, WI, United States; ^4^Department of Physiology and Functional Genomics, University of Florida College of Medicine, Gainesville, FL, United States; ^5^Aging and Metabolism Research Program, Oklahoma Medical Research Foundation, Oklahoma City, OK, United States; ^6^Department of Microbiology, Immunology, and Pathology, Colorado State University, Fort Collins, CO, United States; ^7^Columbine Health Systems Center for Healthy Aging, Colorado State University, Fort Collins, CO, United States

**Keywords:** aging, skeletal muscle, protein synthesis, musculoskeletal, osteoarthritis, animal model, myofiber

## Abstract

Skeletal muscle dysfunction, articular cartilage degeneration, and bone loss occur essentially in parallel during aging. Mechanisms contributing to this systemic musculoskeletal decline remain incompletely understood, limiting progress toward developing effective therapeutics. Because the progression of human musculoskeletal aging is slow, researchers rely on rodent models to identify mechanisms and test interventions. The Dunkin Hartley guinea pig is an outbred strain that begins developing primary osteoarthritis by 4 months of age with a progression and pathology similar to aging humans. The purpose of this study was to determine if skeletal muscle remodeling during the progression of osteoarthritis in these guinea pigs resembles musculoskeletal aging in humans. We compared Dunkin Hartley guinea pigs to Strain 13 guinea pigs, which develop osteoarthritis much later in the lifespan. We measured myofiber type and size, muscle density, and long-term fractional protein synthesis rates of the gastrocnemius and soleus muscles in 5, 9, and 15-month-old guinea pigs. There was an age-related decline in skeletal muscle density, a greater proportion of smaller myofibers, and a decline in type II concomitant with a rise in type I myofibers in the gastrocnemius muscles from Dunkin Hartley guinea pigs only. These changes were accompanied by age-related declines in myofibrillar and mitochondrial protein synthesis in the gastrocnemius and soleus. Collectively, these findings suggest Dunkin Hartley guinea pigs experience myofiber remodeling alongside the progression of osteoarthritis, consistent with human musculoskeletal aging. Thus, Dunkin Hartley guinea pigs may be a model to advance discovery and therapeutic development for human musculoskeletal aging.

## Introduction

Musculoskeletal aging broadly describes the progressive decline in skeletal muscle, bone, tendon, and articular cartilage that contributes to disability, chronic disease, and impaired quality of life in older adults (Roux et al., 2005; Landi et al., 2013; Batsis et al., 2014; Yokota et al., 2015; Williams et al., 2018; Ogawa et al., 2018). One facet of musculoskeletal aging is sarcopenia, which was initially defined as the age-related loss of muscle mass (Rosenberg, 1989). However, loss of muscle mass does not always explain the age-related decline in muscle function (Hughes et al., 2001). Correspondingly, sarcopenia is now broadly used to describe age-related skeletal muscle remodeling and dysfunction (Clark and Manini, 2008; Fielding et al., 2011; Anker et al., 2016; Cruz-Jentoft et al., 2019). Inclusion of functional loss in the definition of sarcopenia is important because skeletal muscle strength is one of the best predictors of all-cause mortality (García-Hermoso et al., 2018). Diagnostic criteria for sarcopenia vary widely but can include a loss in lean muscle mass, a decrease in strength, impaired gait, or impairment in the ability to complete activities of daily living (Fielding et al., 2011). The prevalence of sarcopenia varies extensively due to the lack of consensus criteria for sarcopenia (Bülow et al., 2019a, b; Langer et al., 2019). However, estimates are that 75% of men and 35% of women over the age of 60 years have age-related skeletal muscle dysfunction; these percentages increase to 88% and 53% of men and women over 80 years old (Batsis et al., 2014). Undoubtedly, the projected increase in the aged population will lead to a growing number of individuals living with skeletal muscle dysfunction, and overall musculoskeletal decline (Dhillon and Hasni, 2017), leading to an increased economic burden (Goates et al., 2019). Given both the health and financial burdens, understanding the etiology and discovering therapies to prevent or mitigate age-related skeletal muscle decline, and promote overall musculoskeletal health with aging, is critical.

Musculoskeletal decline is heterogenous and multifactorial in nature. The decline in skeletal muscle is slow and heterogeneous with the average human over the age of 65 losing 1% muscle mass per year (Hughes et al., 2001). Among the disease drivers of aging (López-Otín et al., 2013; Kennedy et al., 2014), inflammation (Schaap et al., 2006), impaired mitochondrial function (Coen et al., 2019), loss of proteostasis (Fernando et al., 2019), and macromolecular damage (Montes et al., 2017) also are intimately linked to musculoskeletal decline. Additional key contributors to development and progression of the muscle aging phenotype include reduced physical activity (Distefano et al., 2018), loss of motor neurons (Hepple, 2018), and resistance to stimuli that should result in muscle protein anabolism (Cuthbertson et al., 2004; Katsanos et al., 2006; Reidy et al., 2017).

Critical to both discovery and translational research to improve human health is availability of laboratory and pre-clinical models that bear close resemblance to the human condition. Musculoskeletal system components interact mechanically, communicate biochemically, and often decline in parallel collectively contributing to falls, fracture risk, and loss of mobility (Bonewald et al., 2013; DiGirolamo et al., 2013). Additionally, evidence suggests that, with advancing age, skeletal muscle dysfunction does not occur in isolation. For example, age-related loss of skeletal muscle strength/function increases the risk for knee osteoarthritis (OA; Lee et al., 2016), and may contribute to knee OA progression (Shorter et al., 2019). Knee OA also imposes a greater risk of developing skeletal muscle dysfunction (Kemmler et al., 2015; Noehren et al., 2018), establishing the two as key musculoskeletal system components that contribute to age-related decline. Therefore, a preclinical model that mimics the systemic age-related musculoskeletal decline in humans is needed.

The Dunkin Hartley guinea pig is a model of spontaneous (also considered primary or idiopathic) OA that closely resembles the onset and disease progression in humans (Jimenez et al., 1997). When fed *ad libitum*, this strain of guinea pig becomes overweight/obese (Radakovich et al., 2019) and begins developing knee OA at approximately 3–4 months of age. By 9 months, there are clear decrements in mobility and, by 18 months, mobility is severely constrained (Santangelo et al., 2014). While the exact cause(s) of OA in this model is still under investigation, inflammation is one factor associated with disease progression (Santangelo et al., 2011). Dunkin Hartley guinea pigs develop OA accompanied by increased systemic inflammation at a relatively early age compared to the lifespan of other outbred strains of guinea pigs (15 months compared to a maximal lifespan of 12 years; Gorbunova et al., 2008). Importantly, an age-related increase in inflammation and oxidative stress (i.e., inflammaging) is also associated with human musculoskeletal aging (Zembron-Lacny et al., 2019). However, age-related skeletal muscle changes have yet to be characterized in these guinea pigs. Thus, we sought to determine if Dunkin Hartley guinea pigs develop a muscle phenotype similar to skeletal muscle from aging humans. The purpose of this study was to determine if skeletal muscle remodeling during the progression of OA in Dunkin Hartley guinea pigs resembles skeletal muscle aging in humans. We evaluated skeletal muscle in Dunkin Hartley guinea pigs and Strain 13 guinea pigs, which served as a comparison model as they develop OA later in their lifespan and have better mobility than Dunkin Hartley guinea pigs (Huebner et al., 2002; Santangelo et al., 2014). If the skeletal muscle phenotype in the Dunkin Hartley guinea pig indeed closely models muscle aging in humans, it may serve as a valuable preclinical model for investigating the etiology of systemic musculoskeletal decline and for evaluating intervention efficacy. Therefore, the purpose of this study was to characterize changes in the skeletal muscle phenotype, including whole muscle composition, myofiber type and size distribution, and rates of muscle protein synthesis, in Dunkin Hartley guinea pigs over a time course encompassing the onset and progression of OA (5, 9, and 15 months of age). We hypothesized that, with age, there would be shifts in the muscle composition, myofiber type and size distribution, and rates of muscle protein synthesis that resemble those that occur during musculoskeletal aging in humans.

## Materials and Methods

### Husbandry, Euthanasia, and Tissue Acquisition

All procedures were approved by the Colorado State University Institutional Animal Care and Use Committee (Protocols 16-6755AA and 19-9129A) and were performed in accordance with the NIH Guide for the Care and Use of Laboratory Animals. 18 male Dunkin Hartley were obtained from Charles River Laboratories (Wilmington, MA, United States) and 18 male Strain 13 guinea pigs were obtained from the United States Army Medical Research Institute of Infectious Diseases (Fort Detrick, MD, United States) at approximately 3.5, 7.5, and 13.5 months of age (mo; *n* = 6 at each age per strain). Animals were maintained at Colorado State University’s Laboratory Animal Resources housing facilities and were monitored daily by a veterinarian. All guinea pigs were singly-housed in solid bottom cages, maintained on a 12–12 h light-dark cycle, and provided *ad libitum* access to food and water. At the time of euthanasia, the guinea pigs were 5, 9, or 15 mo. Two 15 mo Dunkin Hartley guinea pigs had to be urgently euthanized 1 week prior to harvest due to lung and gastrointestinal complications according to the necropsy conducted by a veterinarian. In accordance with the standards of the American Veterinary Medical Association, animals were anesthetized with a mixture of isoflurane and oxygen; immediately afterward, the anesthetized animals were transferred to a chamber filled with carbon dioxide for euthanasia. To confirm euthanasia, bilateral thoracotomy was performed. After euthanasia, a portion of the belly of gastrocnemius and soleus muscles of one leg was excised, mounted, fixed, and frozen in isopentane in OCT for fluorescent immunohistochemistry for myofiber type and size analysis, while the remaining muscle was snap frozen in liquid nitrogen for isotope analysis. We could not collect these measurements for the two premature deaths of the 15 mo guinea pigs. The other leg was formalin fixed with the knee and ankle joints at 90^*o*^ prior to being individually dissected at their attachments to the bone. All limbs were fixed and treated in an identical manner to control for the possibility of fixation-related artifacts. The muscles for this leg were used for volumetric analysis, fiber angle, and immunohistochemistry.

### Skeletal Muscle Mass, Volume, and Density and Tibia Length

Magnetic resonance imaging (MRI) was used to obtain volume in both heads of the gastrocnemius and soleus, which had been formalin fixed with the knee and ankle joints at 90° prior to being individually dissected at their attachments to the bone. MRI scans were performed at Rocky Mountain Magnetic Resonance of Colorado State University, with a 2.3 T Bruker BioSpec, equipped with a 20.5 cm, 100 mT/m gradient system, using a custom built 3.4 cm internal diameter, single channel RF Litz coil (Doty Scientific Inc, Columbia, SC, United States) tuned to detect 1 H at 100.3 MHz. The excised muscles were weighed and then imaged in groups of 8. In the T1- weighted gradient echo images, a fast low-angle shot sequence was used to acquire volumetric images resolved with 0.5 mm isotropic resolution in three-dimensions: echo time = 4.73 ms; repetition time = 15 ms; field of view = 96.0 mm × 33.5 mm × 29.5 mm; matrix size = 192 × 67 × 59.

The volumetric images were exported as DICOM and Analyze 11^§^ was used for segmentation and ROI analysis of muscle volume. Muscle mass and volume were utilized to calculate density – mass divided by volume (mg/mm^3^).

Tibial length was determined using calibrated digital calipers. Measurements were collected on the posterior/caudal aspect of the bone from the intercondylar eminence to the articular surface of the medial malleolus. Measurements were taken in triplicate with the mean recorded.

### Fiber Angle

From the formalin fixed limb, both the medial and lateral gastrocnemius heads were stained in India ink, imaged, and analyzed for fiber angle (θ), defined as the angle of the fiber from the muscle’s line of action (Charles et al., 2016). Measurements were made in four different regions of the muscle using ImageJ and an average angle was recorded. This technique allowed for unbiasing regions of muscle due to the heterogeneity of the gastrocnemius, as previously performed in other rodent muscle (Charles et al., 2016). Measurements were quantified in a blinded and randomized fashion by a single observer. Repeatability testing was completed by performing the measurements twice (*r*^2^ = 0.5953).

### Skeletal Muscle Collagen Content

From the fixed limb and after fiber angle analysis, the gastrocnemius and soleus were paraffin embedded, cross sectioned, and stained with Masson’s Trichrome following an established protocol at the Colorado State University Diagnostic Medicine Center. Masson’s Trichrome staining results in collagen fibers stained blue, nuclei tissue stained black, and background tissue stained red. We imaged the stained cross sections using an upright microscope at 10x magnification and used ImageJ to determine the percentage of area stained blue. To do this, two reviewers set a threshold to quantify the amount of blue present in the cross section and results were subsequently averaged.

Additionally, we used a spectrophotometric method to quantify relative collagen content in tissue using Sirius Red and Fast Green (Chondrex, Inc., Redmond, WA, United States). Portions of the gastrocnemius and soleus were frozen at the time of tissue harvest and 5 μm skeletal muscle cryosections were mounted on microscope slides. Sections were rinsed with 1X phosphate buffered saline (PBS), immersed in the Dye Solution for 30 min at room temperature, and covered to prevent evaporation of the solution. After incubation, we aspirated the solution and then rinsed the tissue section with distilled water. We then applied the Dye Extraction Buffer and then transferred the Buffer to a 96 well plate for spectrophotometric analysis. We measured the OD values at 540 nm and 605 nm and calculated collagenous and non-collagenous using manufacturer instructions.

### Histologic Grading of Osteoarthritis

From the fixed limb, knees were decalcified, sectioned coronally at the level of the medial tibial plateau, and paraffin embedded such that a 5 μm intact central section could be stained with toluidine blue. Following recommended published guidelines, the four anatomic compartments of the joint (medial/lateral tibial plateaus and medial/lateral femoral condyles) were scored using a semiquantitative histopathologic grading scheme (OARSI) such that a total joint score was assigned (Radakovich et al., 2019).

### Skeletal Muscle Fiber Type and Size Distribution

We employed fluorescent immunohistochemistry to measure fiber type and size distribution in the gastrocnemius and soleus muscles. We cryosectioned portions of both the soleus and gastrocnemius that were embedded in OCT and frozen in isopentane cooled by liquid nitrogen during harvest. We then mounted 5 μm skeletal muscle cryosections on microscope slides, allowed them to air dry for 10 min, fixed them in −20°C acetone for 10 min, and rehydrated them in 1X PBS. We then blocked the samples in 10% normal goat serum (NGS) for 1 h and rinsed them in 1X PBS for 30 s. Samples were incubated in the following primary antibodies diluted in 10% NGS for 2 h at room temperature protected from the light: Laminin: Abcam 11576, 1:500; MyHC I: DHSB BA-F8, 1:50; MyHC IIB: DHSB 10F5, 1:50; MyHC IIA: DHSB 2F7 1:50. Following 3, 5 min rinses in 1X PBS, we incubated the cross sections with secondary antibodies also in 10% NGS for 1 h (ThermoFisher AlexaFluor 350 A21093; 647 A21242; 555 A21426; 488 A21121; concentration: 1:500), applied an anti-fade reagent (Prolong Gold Antifade, ThermoFisher), and adhered cover slips to the microscope slides.

The slides were imaged by the Center of Muscle Biology at the University of Kentucky as described (Wen et al., 2018). Briefly, images were acquired using an upright microscope at 20x magnification (AxioImager M1, Zen2.3 Imaging Software; Zeiss, Göttingen, Germany), which automatically acquires consecutive fields in multiple channels. These fields were stitched together in a mosaic image. The different fiber types were visually identified based on color differences in the merged image. The merged images were then analyzed using MyoVision, software developed by the Center for Muscle Biology at the University of Kentucky. The software used the anti-laminin immunofluorescence to establish line and edge structures, generating fiber outlines to provide the cross-sectional area (CSA) of each fiber. Within each fiber, the software then qualified the fiber type based on the fluorescence. Type I fibers were fluorescent at 647 nm, Type IIA fibers at 488 nm, and Type IIB fibers at 555 nm. Fibers that were negative under all channels were classified as Type IIX. An average of over 1000 myofibers were analyzed per animal for each muscle. To analyze fiber size distribution, fibers were categorized into 250 μm^2^ bins. The number of fibers in each bin was then divided by the total number of fibers analyzed to determine the percent distribution of each bin.

### Skeletal Muscle Protein Synthesis

We used the stable isotope deuterium oxide (^2^H_2_O) to measure long-term skeletal muscle protein synthesis rates. 30 days prior to euthanasia, all guinea pigs were given a subcutaneous injection of 0.9% saline enriched with 99% ^2^H_2_O equivalent to 3% of their body weight. Drinking water was then enriched with 8% ^2^H_2_O to maintain deuterium enrichment of the body water pool during the 30-day labeling period. Body water enrichment was determined from plasma as previously described (Robinson et al., 2011; Scalzo et al., 2014; Konopka et al., 2017).

During tissue harvest, approximately 50 mg of gastrocnemius and soleus were collected and frozen immediately in liquid nitrogen. Tissue was later homogenized and fractionated following an established differential centrifugation protocol (Robinson et al., 2011) with one extra step to acquire a collagen-enriched fraction validated by both western blot and proteomic analysis (Miller et al., 2005).

To determine percentage of deuterium enriched alanine skeletal muscle, we followed previously published procedures (Robinson et al., 2011; Drake et al., 2013; Scalzo et al., 2014; Konopka et al., 2017). Approximately 25–50 mg of skeletal muscle was bead homogenized (Next Advance, Inc., Averill Park, NY, United States) in an isolation buffer containing 100 mM KCl, 40 mM Tris–HCl, 10 mM Tris base, 5 mM MgCl2, 1 mM EDTA, and 1 mM ATP (pH 7.5), with phosphatase and protease inhibitors (HALT; ThermoScientific, Rockford, IL, United States). After homogenization, the samples were centrifuged at 800 *g* for 10 min at 4°C. The resulting pellet was enriched with collagen and myofibrillar proteins. The supernatant contained mitochondrial and cytosolic proteins. To separate the collagen and myofibrillar proteins, we isolated and washed the pellet with 500 μL of 100% ethanol and rinsed with 500 μL of Milli-Q water twice. We then added 0.3 M NaOH, incubated for 30 min 37°C, centrifuged at 16,300 *g* for 10 min at 4°C, and transferred the supernatant, which contains myofibrillar proteins, to another tube. We repeated the 0.3 M NaOH incubation to extract any remaining myofibrillar proteins. The remaining collagen pellet was rinsed with Milli-Q water and ethanol. The pellet was then resuspended in 250 μL of 1 M NaOH and placed on a heat block for 15 min at 50°C shaking at 900 rpm. The collagen protein enriched fraction was then incubated in 6 M HCl for 24 h at 120°C for protein hydrolysis.

To precipitate the myofibrillar proteins from the previous supernatant, we added 500 μl of 1 M perchloric acid and centrifuged at 3000 *g*, 4°C for 20 min. After removing the supernatant, we rinsed the pellet with ethanol and Milli-Q water, resuspended in 250 μL of 1 M NaOH, and placed on a heat block for 15 min at 50°C shaking at 900 rpm. The myofibrillar protein enriched fraction was then incubated in 6 M HCl for 24 h at 120°C for protein hydrolysis.

To isolate the mitochondrial and cytosolic proteins, we centrifuged the supernatant from the initial centrifugation at 9000 *g* in 4°C for 30 min. The resulting supernatant, containing cytosolic proteins, was removed and placed in a new tube. The resulting pellet, enriched with mitochondrial proteins, was washed in a buffer containing 100 mM KCl, 10 mM Tris–HCl, 10 mM Tris Base, 1 mM MgCl2, 0.1 mM EDTA, and 1.5% BSA. We then rinsed the pellet in ethanol and Milli-Q water, resuspended in 250 μL of 1 M NaOH, and placed on a heat block for 15 min at 50°C shaking at 900 rpm. The mitochondrial protein enriched fraction was then incubated in 6 M HCl for 24 h at 120°C for protein hydrolysis.

The supernatant containing cytosolic proteins was divided into two 400 μl aliquots. We added 400 μl of 14% sulfosalicylic acid to one aliquot and incubated it on ice for 1 h. After incubation, we centrifuged at 16,000 *g* at 4°C for 10 min, rinsed the pellet with ethanol and Milli-Q water, resuspended the pellet in 250 μL of 1 M NaOH, and placed on a heat block for 15 min at 50°C shaking at 900 rpm. The cytosolic protein enriched fraction was then incubated in 6 M HCl for 24 h at 120°C for protein hydrolysis.

All hydrolysates were ion exchanged, dried in a vacuum, and resuspended in 1 mL of molecular biology grade water. Half of the suspended sample was derivatized by a 1 h incubation of 500 μL acetonitrile, 50 μL K_2_HPO_4_, pH 11, and 20 μL of pentafluorobenzyl bromide. Ethyl acetate was added and the organic layer was removed, dried under nitrogen gas, and reconstituted in 200–600 μL ethyl acetate for analysis on an Agilent 7890A GC coupled to an Agilent 5975C MS as previously described (Robinson et al., 2011; Scalzo et al., 2014; Konopka et al., 2017). The newly synthesized fraction of myofibrillar proteins was calculated from the enrichment of alanine bound in muscle proteins over the entire labeling period, divided by the true precursor enrichment, using the plasma deuterium enrichment over the period of measurement with MIDA adjustment (Busch et al., 2006).

### Statistics

A two-way ANOVA was performed when outcome measures (body mass, muscle mass and density, fiber angle, and protein synthesis) were assessed at all three ages of 5, 9, and 15 mo, to analyze the main effects of both age and strain. If there was significant main effect of age, we conducted a Tukey’s *post hoc* test to specifically compare ages within a strain. Due to tissue availability, we only compared 5 and 15 mo guinea pigs for CSA and fiber type, using Student’s independent *t*-test. To compare CSA distributions, we used Student independent *t*-tests to analyze differences between ages at each 250 μm^2^ bin. GraphPad Prism 8.0 (La Jolla, CA, United States) was used for statistical analysis and to create figures. While statistical significance was set *a priori* at *p* < 0.05, because of the relatively small sample size, we also chose to report values of *p* < 0.10 as noteworthy observations. Data are normally distributed and presented as mean ± standard error when data from individual animals cannot be shown (i.e., CSA distribution).

## Results

### Age Related Changes in Body Mass, Tibia Length, and Joint Degeneration

**FIGURE 1:**
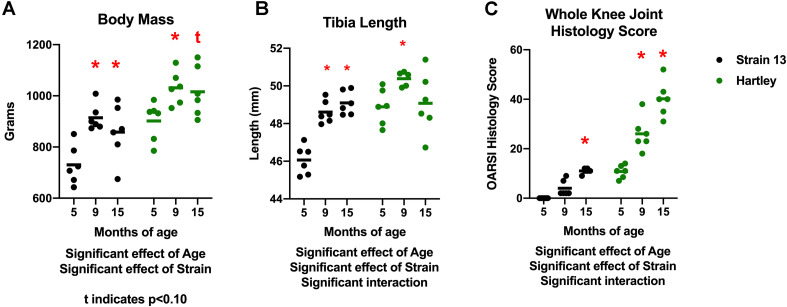
Differences in body mass, tibia length, and whole knee joint histology scores. There was a significant effect of age (*p* < 0.05) on body mass in both Strain 13 and Dunkin Hartley guinea pigs **(A)**. Dunkin Hartley guinea pigs were significantly heavier than Strain 13 guinea pigs. 15 mo Dunkin Hartley guinea pigs tended to be larger than their 5 mo counterparts (*p* = 0.0560), while 15 mo Strain 13 guinea pigs were significantly (*p* < 0.05) larger than 5 mo Strain 13 guinea pigs. However, there was a significant age-related increase in tibia length in Strain 13 guinea pigs (*p* < 0.05) **(B)**. There was a significant effect of age and strain (*p* < 0.05) on whole joint histology scores. Dunkin Hartley guinea pigs displayed a significant increase at 9 and 15 mo in OA severity while Strain 13 guinea pigs only displayed a significant increase at 15 mo **(C)**. *denotes *p* < 0.05 compared to 5 mo. *denotes *p* < 0.05 compared to 5 mo of the same strain; t denotes *p* < 0.10 compared to 5 mo of the same strain.

There was a significant effect of age (*p* < 0.001) on body mass. Body mass increased with age in both strains, with 9 mo guinea pigs weighing significantly more than 5 mo, and 15 mo guinea pigs tending (*p* = 0.06) to weigh more than 5 mo (Figure 1A). There was no difference in body mass between 9 and 15 mo animals in either strain, indicating that growth plateaued prior to 9 mo of age. Correspondingly, there was an increase in tibia length with age (*p* < 0.05), although this was observed predominantly in Strain 13 (interaction effect *p* < 0.05; Figure 1B). Tibia length did not differ between 9 and 15 mo animals, again reflecting that growth plateaued by 9 mo. Both strains experienced an age-related increase in OA severity as assessed by histologic grading (age effect *p* < 0.05). Dunkin Hartley guinea pigs had more severe OA than Strain 13 counterparts (strain effect *p* < 0.05; Figure 1C). Notably, the average knee joint score of 15 mo Strain 13 guinea pigs was no different than 5 mo Dunkin Hartley guinea pigs.

### Age Related, Muscle-Specific, and Strain-Specific Differences in Muscle Mass and Density

**FIGURE 2:**
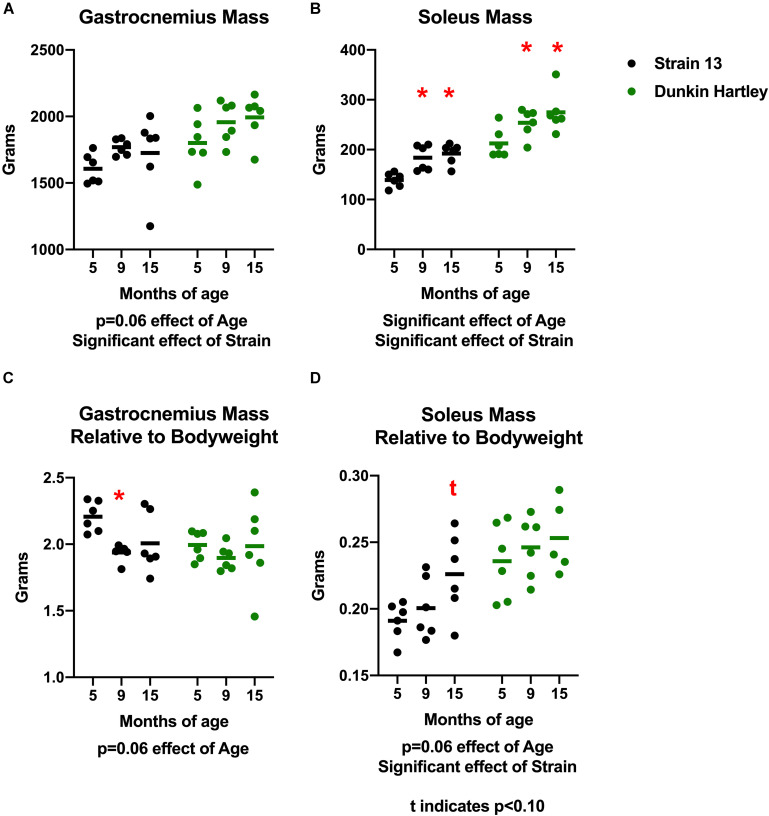
Differences in gastrocnemius and soleus mass and mass relative to bodyweight. There was non-significant effect of age on gastrocnemius mass **(A)** and a significant effect on soleus mass **(B)**. When expressed relative to body mass, there was a non-significant effect of age in both the gastrocnemius **(C)** and soleus **(D)**. *Post hoc* analysis revealed no differences in relative muscle mass between the Dunkin Hartley guinea pigs at any age. *denotes *p* < 0.05 compared to 5 mo, t denotes *p* < 0.05 compared to 5 mo.

To determine whether or not there were muscle-specific declines concomitant with the progression of OA, we measured skeletal muscle mass, volume, and density in the hindlimb of the guinea pigs. There was a non-significant positive effect of age on gastrocnemius mass (*p* = 0.06) in both strains of guinea pigs. Dunkin Hartley guinea pigs had larger gastrocnemii than Strain 13 guinea pigs (*p* < 0.05; Figure 2A). There was a significant effect of age (*p* < 0.05) on soleus mass, with 15 mo guinea pigs having greater (*p* < 0.05) mass than their 5 mo counterparts (Figure 2B). However, there were no differences in either the gastrocnemius or soleus between 9 mo and 15 mo guinea pigs. Similar to the gastrocnemius, Dunkin Hartley guinea pigs had greater soleus mass than Strain 13 guinea pigs (*p* < 0.05).

Because there was a significant effect of age on body mass, we also expressed muscle mass relative to body mass. There was a non-significant, negative effect of age on gastrocnemius mass relative body mass (*p* = 0.06) accounted for by Strain 13 guinea pigs (Figure 2C). However, there was a non-significant, positive effect of age (*p* = 0.06) on soleus mass relative to body mass (Figure 2D). Dunkin Hartley guinea pigs did have significantly greater soleus mass relative to body mass compared to Strain 13 guinea pigs (Figure 2D). However, there were no significant differences between any age in either the gastrocnemius (Figure 2C) or soleus (Figure 2D) of the Dunkin Hartley guinea pigs.

**FIGURE 3:**
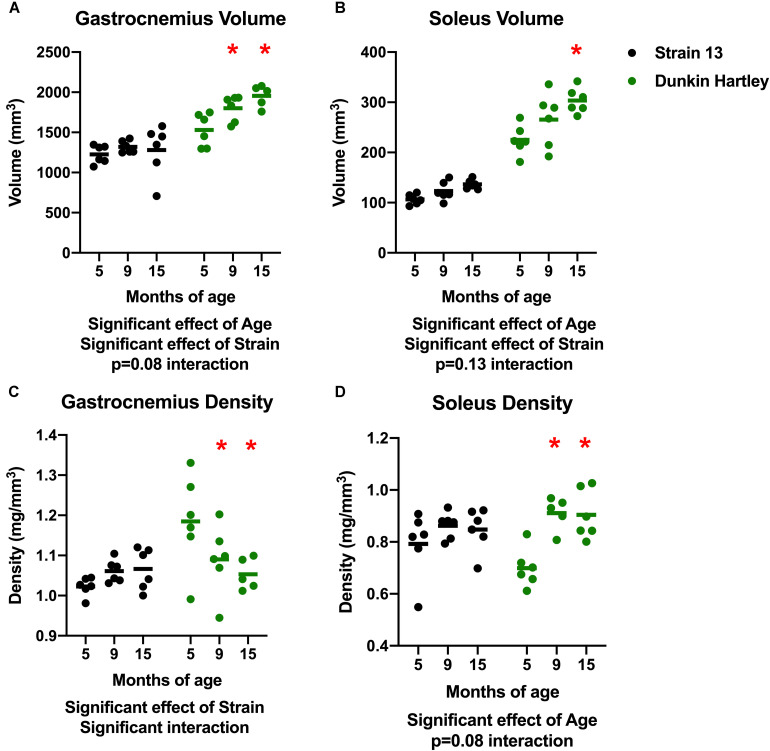
Differences in muscle volume and density. There was a significant, positive effect of age on gastrocnemius **(A)** and soleus **(B)** muscle volume, though there was a non-significant interaction in the gastrocnemius, suggesting the increase in volume primarily occurred in Dunkin Hartley guinea pigs. Gastrocnemius density was significantly lower in 9 and 15 mo Dunkin-Hartley guinea pigs compared to 5 mo counterparts, whereas there was no differences observed in Strain 13 guinea pigs **(C)**. In the soleus, muscle density was significantly greater in 9 and 15 mo Dunkin-Hartley guinea pigs compared to 5 mo **(D)**. *denotes *p* < 0.05 compared to 5 mo.

There was a significant effect of age on gastrocnemius volume, with volume increasing with age (Figure 3A). Similarly, there was a significant increase in soleus volume with age (Figure 3B). There were, however, no differences in volume of either the gastrocnemius or soleus in Strain 13 guinea pigs. There was a significant, negative effect of age on gastrocnemius density, with lower density in 15 mo Dunkin Hartley guinea pigs compared to 5 mo (*p* < 0.05; Figure 3C). However, this effect was not observed in Strain 13 guinea pigs. There was a significant main effect of age on the soleus density in Dunkin Hartley guinea pigs (*p* < 0.001), but again, this was not observed in Strain 13 guinea pigs (Figure 3D).

### Age-Related Changes in Myofiber Cross Sectional Area, Size, Distribution, and Fiber Type Composition

**FIGURE 4:**
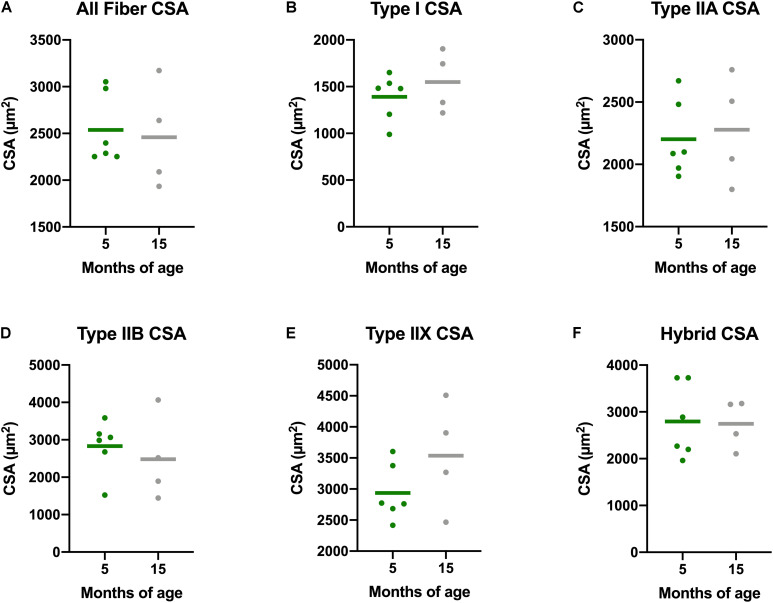
Muscle fiber type-specific CSA averages in the gastrocnemius of Dunkin Hartley guinea pigs. There were no differences between the CSA of skeletal muscle fibers of any type in the gastrocnemii of 5 mo and 15 mo Dunkin Hartley guinea pigs **(A–F)**.

There is a decline in myofiber CSA in addition to a greater proportion of type I, or slow, myofibers in aging adults. Accordingly, we measured myofiber CSA and fiber type in both the gastrocnemius and soleus of these guinea pigs. Average CSA of all gastrocnemius myofibers of Dunkin Hartley guinea pigs did not change as a consequence of age (Figure 4). Similarly, there were no age-related differences when mean CSA was measured within each fiber type of the gastrocnemius. We also saw no difference in myofiber CSA in the soleus of Dunkin Hartley guinea pigs (Supplementary Figure 1A). There was a significant (*p* < 0.05) difference in soleus myofiber CSA between 5 mo and 15 mo Strain 13 guinea pigs (Supplementary Figure 1A), but not in the gastrocnemius (Supplementary Figure 2).

**FIGURE 5:**
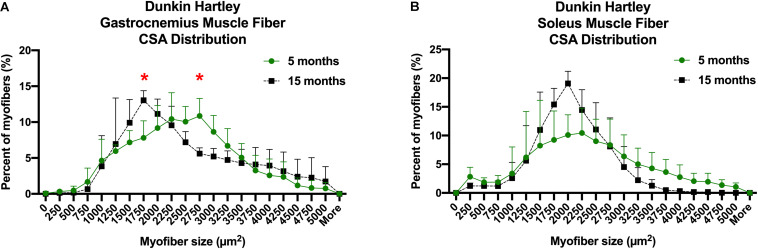
CSA distribution of all myofibers in both the gastrocnemius and soleus of Dunkin-Hartley guinea pigs. 15 mo guinea pigs had a greater proportion of 1750 μm^2^ myofibers and less 2750 μm^2^ myofibers compared to 5 mo guinea pigs (*p* < 0.05; **A)**. However, there was no difference in myofiber size distribution in the soleus **(B)**. *denotes *p* < 0.05 compared to 5 mo.

**FIGURE 6:**
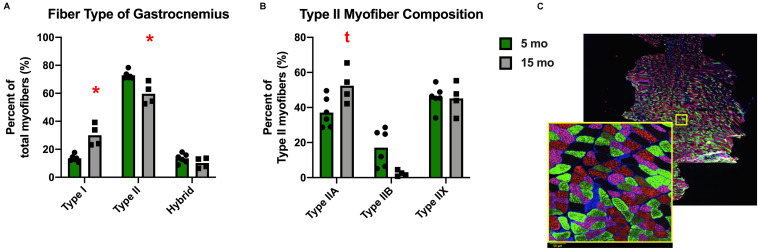
Fiber type composition of the gastrocnemius of Dunkin Hartley guinea pigs. There was a significant greater amount of type I myofibers and lesser amount of type II myofibers in 15 mo Dunkin Hartley guinea pigs compared to 5 mo guinea pigs **(A)**. There is a trend (*p* = 0.07) for less type IIB myofibers in 15 mo guinea pigs as well **(B)**. The example of fiber typing includes a zoomed in portion to show detail. Laminin was fluorescent at 350 nm, type I fibers were fluorescent at 647 nm, type IIA fibers at 488 nm, and type IIB fibers at 555 nm. Fibers that were negative under all channels were classified as type IIX. Scale bar (bottom left) = 50 μm. **(C)**. *denotes *p* < 0.05 compared to 5 mo; t denotes *p* < 0.10 compared to 5 mo.

Despite the lack of difference in the average myofiber CSA, there was a change in fiber size distribution in the gastrocnemius of Dunkin Hartley guinea pigs. 15 mo guinea pigs had a greater proportion of 1750 μm^2^ myofibers and less 2750 μm^2^ myofibers compared to 5 mo guinea pigs (*p* < 0.05; Figure 5A). In the soleus, there was no change in myofiber size distribution (Figure 5B). There was no difference in myofiber size distribution of 15 mo Strain 13 guinea pigs compared to 5 mo counterparts in the gastrocnemius (Supplementary Figure 3A). However, 15 mo had a greater proportion of myofibers of 2750 μm^2^ in the soleus (Supplementary Figure 3B). While there were no differences in CSA in any fiber types in the Dunkin Hartley guinea pigs (Figure 4), 15 mo Dunkin Hartley guinea pigs had a significantly lower proportion type II myofibers and greater proportion of type I myofibers in the gastrocnemius compared to 5 mo (Figure 6A). The lower proportion of type IIB myofibers seems to have accounted for the difference in fiber type composition (*p* = 0.07; Figure 6B). While we did not observe a difference in type II myofiber content in 15 mo Strain 13 guinea pigs (Supplementary Figure 4), there was a significantly greater proportion of type I myofibers in the gastrocnemius compared to 5 mo counterparts (Supplementary Figure 4).

### Skeletal Muscle Architecture and Collagen Content

**FIGURE 7:**
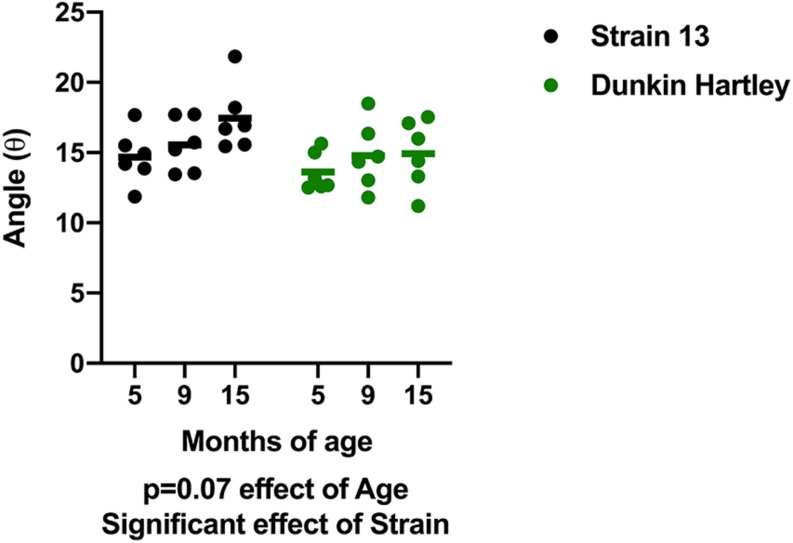
Fiber angle of the gastrocnemius. There was a non-significant increase in fiber angle with age in guinea pigs (*p* = 0.0740). Dunkin-Hartley guinea pigs had a lower fiber angle than Strain 13 guinea pigs.

**FIGURE 8:**
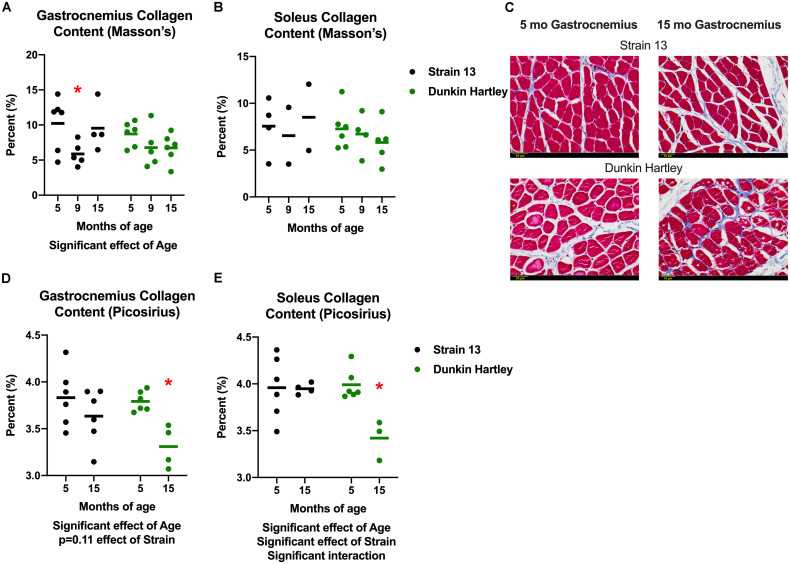
Collagen content in the guinea pig skeletal muscle. As assessed with Masson’s Trichrome staining, there was a significant age-related decrease in collagen content in the gastrocnemius **(A)**, but not the soleus **(B)**. Representative images of stained 5 and 15 mo gastrocnemius cross sections **(C)**. Assessed with spectrophotometry, collagen content was significantly lower in both the gastrocnemius **(D)** and soleus **(E)** of 15 mo Dunkin Hartley guinea pigs compared to 5 mo guinea pigs (*p* < 0.05). Scale bar (bottom left) = 50 μm. *denotes *p* < 0.05 compared to 5 mo.

In addition to changes in skeletal muscle size, humans also experience changes in skeletal muscle structure that impair whole muscle contractile strength. Therefore, we measured fiber angle and collagen content in guinea pig skeletal muscle. There was a non-significant, age-related increase (*p* = 0.07) in gastrocnemius fiber angle (Figure 7). Dunkin Hartley guinea pigs had a significantly smaller fiber angle than Strain 13 guinea pigs. Using Masson’s Trichrome staining, there was a significant age-related decrease in collagen content in the gastrocnemius, though this difference was predominantly observed between 5 mo and 9 mo Strain 13 guinea pigs (Figure 8A). There were no age-related differences in soleus collagen content (Figure 8B). Results of the spectrophotometric picosirius assay of collagen content, however, indicated that 15 mo guinea pigs had lower collagen content than 5 mo guinea pigs in both the gastrocnemius (*p* < 0.05; Figure 8D) and soleus (*p* < 0.05; Figure 8E), and this was only observed in Dunkin Hartley guinea pigs.

### Skeletal Muscle Protein Synthesis

**FIGURE 9:**
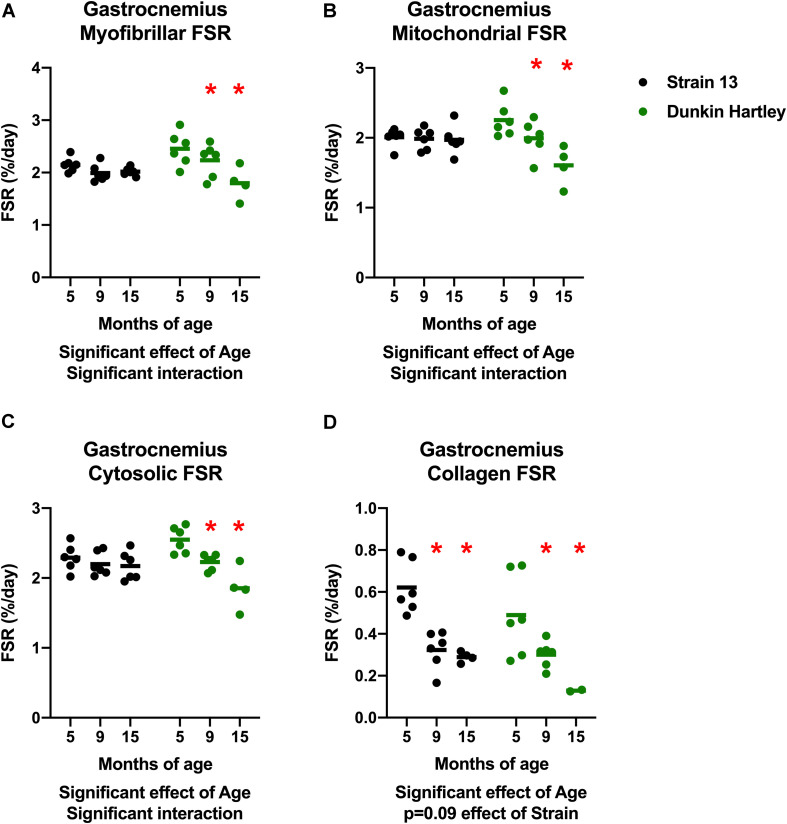
Skeletal muscle protein synthesis rates in the gastrocnemius. In all subfractions of the gastrocnemius there was a significant, negative effect (*p* < 0.05) of age on fractional synthesis rates. In all subfractions except collagen, this age-related decline was present only in Dunkin Hartley guinea pigs **(A–C)**. In the collagen subfraction, there was an age-related decline in FSR in both Strain 13 and Dunkin-Hartley guinea pigs **(D)**. *denotes *p* < 0.05 compared to 5 mo. *n* = 2 for DH 15 mo gastrocnemius collagen due to no enrichment levels detected by GC/MS for two samples.

An imbalance in protein turnover (i.e., the balance between protein synthesis and breakdown) contributes to the age-related decline in skeletal muscle mass. Accordingly, we measured to skeletal muscle protein synthesis rates using the stable isotopic tracer ^2^H_2_O. In the gastrocnemius, myofibrillar, cytosolic, and mitochondrial fractional synthesis rates (FSR) were all lower in 15 mo guinea pigs compared to 5 mo in Dunkin Hartley guinea pigs only (*p* < 0.05; Figures 9A–C). In the Dunkin Hartley guinea pigs, myofibrillar protein synthesis was lower in 15 mo compared to 5 mo guinea pigs (*p* < 0.05; Figure 9A). Cytosolic and mitochondrial FSR were significantly lower at each time point in Dunkin Hartley guinea pigs (Figures 9B,C). There was an age-related decrease (*p* < 0.05) in collagen FSR in both Strain 13 and Dunkin Hartley guinea pigs (Figure 9D).

**FIGURE 10:**
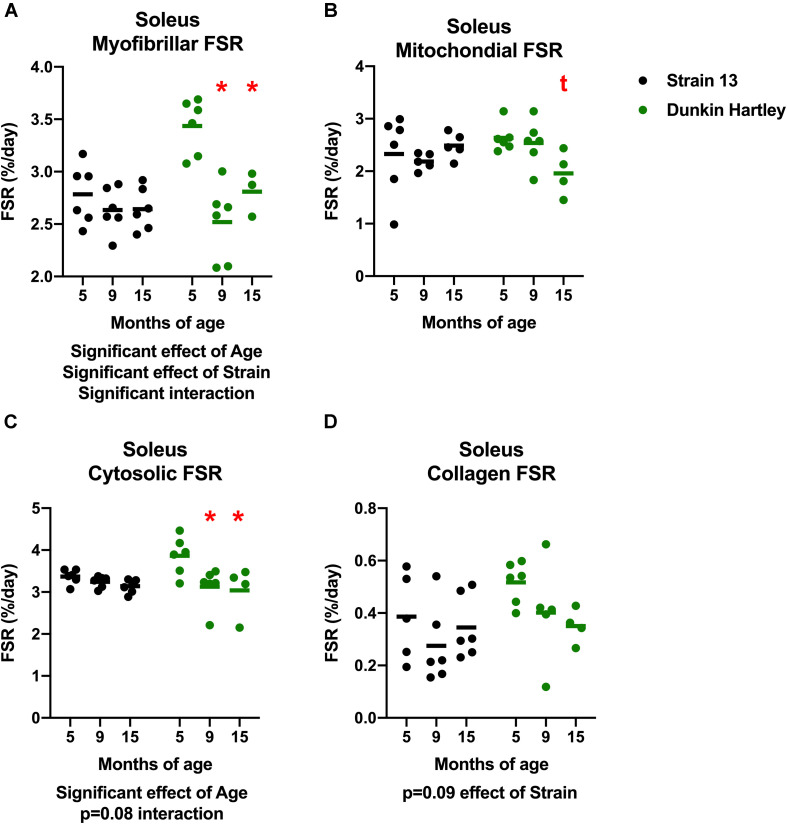
Skeletal muscle protein synthesis rates in the soleus. There was a significant, negative effect (*p* < 0.05) of age on fractional synthesis rates in the myofibrillar **(A)** and cytosolic **(C)** subfractions of the soleus, with 15 mo Dunkin Hartley guinea pigs having significantly slower rates of protein synthesis than 5 mo counterparts. Mitochondrial FSR of the soleus was non-significantly (*p* = 0.0666) slower in 15 mo Dunkin Hartley guinea pigs compared to 5 mo Dunkin Hartley guinea pigs **(B)**. However, there was no effect (*p* > 0.10) of age on collagen FSR **(D)**. *denotes *p* < 0.05 compared to 5 mo; t denotes *p* < 0.10 compared to 5 mo. *n* = 3 for DH 15 mo soleus myofibrillar FSR due to no enrichment levels detected by GC/MS in one sample.

Similar to the gastrocnemius, soleus synthesis rates were slower in the older Dunkin Hartley guinea pigs in each fraction except for collagen (Figure 10). Myofibrillar FSR was lower in the 9 mo and 15 mo compared to the 5 mo Dunkin Hartley guinea pigs (*p* < 0.05; Figure 10A). In Dunkin Hartley guinea pigs, cytosolic FSR was significantly lower in 9 mo and 15 mo compared to 5 mo guinea pigs (Figure 10B). Mitochondrial FSR was non-significantly lower in 15 mo Dunkin Hartley guinea pigs compared to their 5 mo counterparts (*p* = 0.07; Figure 10C). No age-related differences in myofibrillar, mitochondrial, or cytosolic FSR were observed in Strain 13 guinea pigs. There were also no significant age-related differences in collagen protein synthesis in the soleus of either Strain 13 or Dunkin Hartley guinea pigs (Figure 10D).

## Discussion

Musculoskeletal decline is a key contributor to disability (Janssen et al., 2002) and cardiometabolic disease (Hunter et al., 2019), as well as increases the risk of mortality independent of age (Pasco et al., 2017). Obtaining a full understanding of mechanisms contributing to musculoskeletal decline is hampered by complex contributions from systemic components including skeletal muscle, bone, articular cartilage, and tendon, and by the relative absence of preclinical models that closely and comprehensively model the human musculoskeletal aging phenotype. The overall goal of this study was to characterize changes in the skeletal muscle phenotype during a time course that encompasses the onset and progression of joint degeneration in Dunkin Hartley guinea pigs, a model of idiopathic OA. Our findings document, for the first time, evidence of skeletal muscle remodeling in Dunkin Hartley guinea pigs that is consistent with those observed during musculoskeletal aging in humans. Moreover, the lack of changes observed in the comparison Strain 13 group, which is not prone to developing OA this early in the lifespan, suggests that the predominance of the observed age-related skeletal muscle changes is unique to the Dunkin Hartley guinea pigs. During this 10-month period, we observed a decline in type II myofibers, a shift in fiber size distribution favoring smaller muscle fibers, progressive declines in muscle protein synthesis rates, and a decline in gastrocnemius density, which are all observed in human skeletal muscle aging. Moreover, these skeletal muscle changes are accompanied by impairments in other components of the musculoskeletal system, specifically degeneration of articular cartilage (Jimenez et al., 1997). The current study, combined with previous work detailing progression of joint degeneration and inflammation (Santangelo et al., 2011), suggest that Dunkin Hartley guinea pigs may serve as a valuable model of systemic musculoskeletal decline.

### Similarities Between Skeletal Muscle Remodeling in Dunkin Hartley Guinea Pigs and Aging Humans

Over the time course of the current study, we observed that Dunkin Hartley guinea pigs have a change in muscle fiber size distribution favoring smaller fibers, fiber type composition shifting toward type I and IIA fibers, lower muscle density, and slower rates of muscle protein synthesis. Collectively, these changes suggest skeletal muscle remodeling that is consistent with the aged skeletal muscle phenotype in humans. Interestingly, these observations occurred by an age (15 mo) that is young relative to the maximal predicted lifespan of other strains of guinea pigs [∼10% of the predicted 12 years (Gorbunova et al., 2008)]. The observed changes in skeletal muscle in this study, along with progression of joint degeneration at a young age relative to predicted species maximal lifespan, presents both strengths and potential limitations as a preclinical model to understand musculoskeletal decline. First, the Dunkin Hartley guinea pig could be valuable to test underlying mechanisms and therapeutic targets to combat age-related skeletal muscle derangements over a short time period. Second, the skeletal muscle remodeling observed in the current study occurred within 10 months, which offers a convenient model to test long-term interventions that might modulate the progression of skeletal muscle decline. In comparison, murine models only begin demonstrating signs of muscle aging well after 20 months of age, much closer to their maximal predicted lifespan [50% of 40 months (Graber et al., 2015; Strong et al., 2016)].

A potential limitation worth noting is that there is growth between the 5 and 9 mo time points. While this is not entirely reflective of human musculoskeletal aging, humans do experience an increase in bodyweight, primarily in adiposity, with age (Batsis et al., 2015). Guinea pigs reach sexual maturity by 2.5 mo and in this study both body mass and muscle mass plateaued by 9 mo (Figures 1,2). Importantly, we observed declines in protein synthesis between 9 and 15 mo of age independent of growth (Figures 9, 10). The continued decline in protein synthesis is, therefore, likely a characteristic of musculoskeletal remodeling associated with overall musculoskeletal decline in the Hartley strain, given a lack of difference in the Strain 13 guinea pigs. Though we did not measure myofiber CSA or distribution in 9 mo guinea pigs, 15 mo Dunkin Hartley guinea pigs had a greater percentage of smaller and slow twitch myofibers which are both observed in older adult humans (Spendiff et al., 2016; Holloway et al., 2018). These myofiber changes by 15 mo indicate that any increases in myofiber CSA that occurred during the period of growth (i.e., from 5 to 9 mo) were lost during OA progression and aging (i.e., from 9 to 15 mo). In future studies, we will focus on mechanisms of musculoskeletal decline after growth plateaus (∼9 mo) to better understand the phenotypic shifts and skeletal muscle strength, that are both consequences of musculoskeletal aging. Moreover, we aim to understand whether or not interventions, such as calorie restriction (Radakovich et al., 2019), may ameliorate the progression of the aged musculoskeletal phenotype we observed in these guinea pigs.

In humans, muscle with mixed fiber types (such as the gastrocnemius or vastus lateralis) are more susceptible to loss of mass compared to muscles comprised primarily type I muscle fibers, such as the soleus, which are generally unaffected by age (Larsson et al., 1978; Lexell et al., 1988). This is likely related to the fact that sarcopenia is associated with the loss of larger, type II muscle fibers and an increase in type I muscle fibers (Nilwik et al., 2013). In humans, type II myofibers atrophy and type I myofibers remain largely unchanged resulting in a shift in myofiber size distribution toward smaller myofibers (Nilwik et al., 2013; Spendiff et al., 2016). We observed the same phenomenon in the gastrocnemius of Dunkin Hartley guinea pigs. From 5 months to 15 months of age, there was a shift toward smaller myofibers. Moreover, there was a lower percentage of type II myofibers and a greater percentage of type I myofibers. These age- and OA-related differences, except for the increased proportion of type I myofibers in the gastrocnemius, were not observed in the Strain 13 guinea pigs. More research is necessary to determine if these changes in muscle composition and myofiber size result in loss of strength and other functional decrements as observed in aging humans (Nilwik et al., 2013).

As humans age, there is generally an increase in fat deposition within (intramuscular triglycerides, IMTG) or around (intermuscular adipose tissue, IMAT) skeletal muscle. Only in Dunkin Hartley guinea pigs did we observe a lower gastrocnemius density in 15 mo guinea pigs compared to 5 mo counterparts (Figure 3C). The only logical explanation for this decreased density is greater fat deposition given that fat has lower density than muscle. However, there was an age-related increase in soleus density between young and older Dunkin Hartley guinea pigs (Figure 3D). The contrasting outcomes in gastrocnemius and soleus densities in young and older guinea pigs could be explained by the fact that type II fibers are at greater risk of lipid spillover due to an inability to switch to from carbohydrate to lipid oxidation (i.e., metabolic inflexibility; Carter et al., 2019). Both IMTG (Coen and Goodpaster, 2012; Rivas et al., 2016) and IMAT (Goodpaster et al., 2001; Scott et al., 2015; Konopka et al., 2018) are negatively associated with skeletal muscle function. It remains unclear, however, if lower density in gastrocnemius is associated with greater concentrations of specific lipid species that impart deleterious effects on skeletal muscle function.

Few published studies include data focused on the influence of age on collagen content in human skeletal muscle. Most suggest there is no change in collagen content with age in humans (Babraj et al., 2005; Haus et al., 2007; Kragstrup et al., 2011), a decrease in collagen turnover (Babraj et al., 2005), and an accumulation of oxidative damage (Haus et al., 2007). In our guinea pigs, the two methods we employed to measure collagen content demonstrated either no difference or lower collagen levels in 15 mo compared to 5 mo guinea pigs (Figure 8). However, there was a precipitous decline in muscle collagen synthesis rate with age (Figures 9D, 10D). Accordingly, collagen breakdown must have sharply declined for there to be similar collagen content. A decline in collagen protein turnover would likely have led to the accumulation of oxidatively damaged/crosslinked collagen and imparted decrements in function as is the case in in humans (Haus et al., 2007). However, further work is needed to measure the age-related differences in oxidatively damaged collagen proteins and muscle strength in these guinea pigs.

In humans, fiber angle has been shown to decrease in the gastrocnemius and soleus with age, which is likely due to decreases of sarcomeres both in series and in parallel (Morse et al., 2005). We observed an age-related increase in fiber angle in the gastrocnemius of both strains (Figure 7), but this could be due to continued body growth between the ages of 5 and 9 mo (Figures 1, 2), and reflect greater muscle size between 5 and 15 mo. These are the first measures to be assessed cross-sectionally in these guinea pig strains, but are similar to those reported in other strains (Powell et al., 1984). Future studies should address the potential relationship between fiber angle and force production and how they change with in this model of musculoskeletal decline.

Humans experience a decline in skeletal muscle mass at a relatively slow rate (∼1% decline per year; Reid et al., 2014) beginning later in the lifespan. In order for there to be a loss of muscle mass, protein breakdown rates must exceed protein synthesis rates. The literature is equivocal on whether or not protein breakdown changes with age (Volpi et al., 2001; Aas et al., 2019) which could be a consequence of relying on static markers. Resting protein synthesis between young and older adults appears not to be different (Volpi et al., 2001). However, with aging there is an impairment in protein synthesis in response to anabolic stimuli such as protein ingestion and exercise (Cuthbertson et al., 2004; Brook et al., 2016; Rivas et al., 2016). As a result, cumulative protein synthesis rates in human skeletal muscle likely decline very slowly with age (Reid et al., 2014). This is in contrast to other rodent models, such as mice and rats, that experience abrupt and sharp changes in protein turnover (Rennie et al., 2010). Using the stable-isotopic tracer deuterium (^2^H_2_O), we measured the cumulative protein synthesis over 30 days, capturing both resting and anabolic episodes of protein synthesis in these guinea pigs. 15 mo Dunkin Hartley guinea pigs had lower rates of protein synthesis in all the subfractions of both gastrocnemius and soleus muscles compared to 5 mo counterparts (Figures 9, 10). In comparison, Strain 13 guinea pigs maintained FSR except for the collagen subfraction (Figures 9, 10). Thus, the decline in FSR observed in Dunkin Hartley guinea pigs may be a consequence of the age-related progression of overall musculoskeletal decline. Interestingly, using a similar isotope labeling approach, we recently reported that the Fisher344 Brown Norway (F344BN) rat strain experiences an age-related increase, not decrease, in cumulative skeletal muscle protein synthesis rates between 24 mo, 28 mo, and 30 mo (Miller et al., 2019). This discrepancy in age-related changes in protein synthesis may be influenced by a number of factors including the labeling period (Miller et al., 2015), the rates of protein degradation, and species differences. Regardless, our data support that aging disrupts skeletal muscle protein turnover and this age-related perturbation contributes to overall impairments in protein homeostasis, a well-established driver of aging (López-Otín et al., 2013; Kennedy et al., 2014).

A recent study leveraging ^2^H_2_O methods to facilitate long-term assessment of protein synthesis rates found no difference in myofibrillar FSR between young and older adults prior to exercise training, but an age-related impairment in the response to training (Brook et al., 2016). In our study, we found that the declines in FSR (i.e., myofibrillar and mitochondrial subfraction) are consistent with the smaller myofiber size distribution observed through immunohistochemistry as well as lower gastrocnemius muscle density. While statistically significant, the difference in FSR between 5 and 15 mo guinea pigs is small (<1%), which may reflect small differences in the anabolic response to activity and/or feeding and may contribute the similar slow and progressive decline in muscle protein synthesis observed in human musculoskeletal aging.

### Potential Mechanisms Underlying the Skeletal Muscle Remodeling in Dunkin Hartley Guinea Pigs

At the moment, there is no clear, established mechanism for the early onset of the skeletal muscle aging phenotype we observed in the Dunkin Hartley guinea pigs. This strain of guinea pig develops primary OA that histologically resembles human OA (Jimenez et al., 1997). Strain 13 guinea pigs do not develop OA this early in their lifespan and did not exhibit most of the age-related differences in skeletal muscle we observed in the Dunkin Hartley guinea pigs. This contrast between strains suggests that OA may influence, or be reflective of, the overall musculoskeletal decline in the Dunkin Hartley guinea pigs. The coexistence of joint degeneration and skeletal muscle remodeling similarly exists in human musculoskeletal aging. However, it is difficult to discern a causal relationship between the two. In humans, knee OA increases the risk for sarcopenia (Kemmler et al., 2015), which may be at least in part due to diminished levels of physical activity (Herbolsheimer et al., 2016). However, we did not monitor spontaneous physical activity in these guinea pigs. Physical activity is known to influence skeletal muscle mass, quality (e.g., fiber type, density), and function (Ubaida-Mohien et al., 2019). Given that knee OA generally decreases physical activity (Miller et al., 2001), we predict that decreases in physical activity may account for some of the skeletal muscle remodeling we observe at a relatively young age in these guinea pigs.

Another mechanism that could explain the early onset muscle aging phenotype is increased systemic inflammation. Compared to a strain of guinea pig that is not prone to primary OA at a young age, Dunkin Hartley guinea pigs have higher levels of the pro-inflammatory mediator interleukin-1β (IL-1β; Santangelo et al., 2011). Interventions such as caloric restriction (Bendele and Hulman, 1991; Radakovich et al., 2019), elicit lower levels of systemic inflammatory markers compared to *ad libitum* fed controls and delay the onset of OA in this model. In humans, both knee OA and elevated levels of inflammatory mediators are associated with sarcopenia (Schrager et al., 2007; Shorter et al., 2019). Additionally, age-related increases in IL-1β are associated with decrements in peak knee extension torque (Zembron-Lacny et al., 2019). Inflammation is a known disease driver of aging (Franceschi and Campisi, 2014; Kennedy et al., 2014) and implicated in most age-related chronic diseases (Ferrucci and Fabbri, 2018). Therefore, future inquiry will include inflammatory profiling systemically as well as locally in skeletal muscle and other tissues beyond the musculoskeletal system in these guinea pigs to determine if they are a more encompassing model of inflammaging.

### Comments, Conclusions, and Future Directions

Laboratory models, by definition, do not provide perfect replications of human clinical conditions. Much of what is currently known about skeletal muscle aging comes from research using models designed to study muscle disuse in either rodents or humans (Morey-Holton et al., 2005; Rudrappa et al., 2016), which have yielded promising insight into mechanisms and therapeutics (Ebert et al., 2019), or models of muscle denervation (Siu and Alway, 2009). Additional insights about potential contributors to muscle loss come from genetic models including the PolgA mutant mouse that develops a frail phenotype (Trifunovic et al., 2004; Hiona et al., 2010, Romanick et al., 2013). Developing a model that more closely mimics the complexity of human disease, in this case the systemic musculoskeletal decline that accompanies advancing age, is important for maximizing success in translational research (Denayer et al., 2014). Collectively, our results suggest that Dunkin Hartley guinea pigs could be a valuable addition to the models currently being used for both discovery and translational research in musculoskeletal aging. Within a relatively short period of time, this unique non-transgenic, outbred model develops aspects of systemic human musculoskeletal aging including progressive inflammation (i.e., inflammaging), joint degeneration (Santangelo et al., 2011, 2014), evidence of declining bone strength (unpublished observations), and the alterations in skeletal muscle phenotype reported here.

While we assessed a number of well-recognized aspects of skeletal muscle remodeling associated with human aging, there are other important components of the aging muscle phenotype we have yet to measure in this unique guinea pig model, most notably muscle function. We predict that the changes in muscle phenotype we observed here would be paralleled by declines in muscle function. The observed loss of type IIB fibers is known to be a key determinant of muscle strength (Nilwik et al., 2013). Furthermore, myofiber size distribution favoring a larger percentage of smaller fibers is also a characteristic of older adults and has potential to impart decreased muscle force production (Spendiff et al., 2016). We have not yet measured changes in spontaneous physical activity, but anticipate doing so in future studies with the prediction that Dunkin Hartley guinea pigs likely model the age-related decline in physical activity typically observed in humans. Dunkin Hartley guinea pigs do experience decrements in gait (i.e., slower gait speed, shorter stride length) compared to Strain 13 guinea pigs, which could be reflective of impaired neuromuscular and musculoskeletal function (Santangelo et al., 2014). It is important to note that the current observations were only in male guinea pigs. Given that the onset and prevalence of decline in muscle function differs between aging males and females (Frontera et al., 2000), investigating the age-related changes in skeletal muscle in female guinea pigs is another important focus of our follow-up studies. We posit that the musculoskeletal decline we have comprehensively characterized in Dunkin Hartley guinea pigs, will provide a valuable platform for contributing to the evolving understanding of human musculoskeletal aging and, hopefully, interventions to improve mobility, independence, and quality of life for aging humans.

## Data Availability Statement

The original contributions presented in the study are included in the article/Supplementary Material, further inquiries can be directed to the corresponding author.

## Ethics Statement

The animal study was reviewed and approved by Colorado State University Institutional Animal Care and Use Committee.

## Author Contributions

This study was conducted at Colorado State University. KH, KS, and RR contributed to the conception and design. All authors contributed to data acquisition and/or interpretation of data. RM and MW wrote the first manuscript draft. All authors critically revised the manuscript and provided intellectual and approved the final version of the manuscript submitted for publication.

## Conflict of Interest

The authors declare that the research was conducted in the absence of any commercial or financial relationships that could be construed as a potential conflict of interest.
