# Supplementary material for: The Dunkin Hartley Guinea Pig Is a Model of Primary Osteoarthritis That Also Exhibits Early Onset Myofiber Remodeling That Resembles Human Musculoskeletal Aging
Source: Front Physiol. 2020 Oct 29;11:571372. doi: 10.3389/fphys.2020.571372 (PMC7658338; doi:10.3389/fphys.2020.571372)
Supplement: Supplementary file 1 [file Table_1.DOCX]

**The Dunkin Hartley guinea pig is a model of primary osteoarthritis that also exhibits early onset myofiber remodeling that resembles human musculoskeletal aging**

Supplementary Material

##
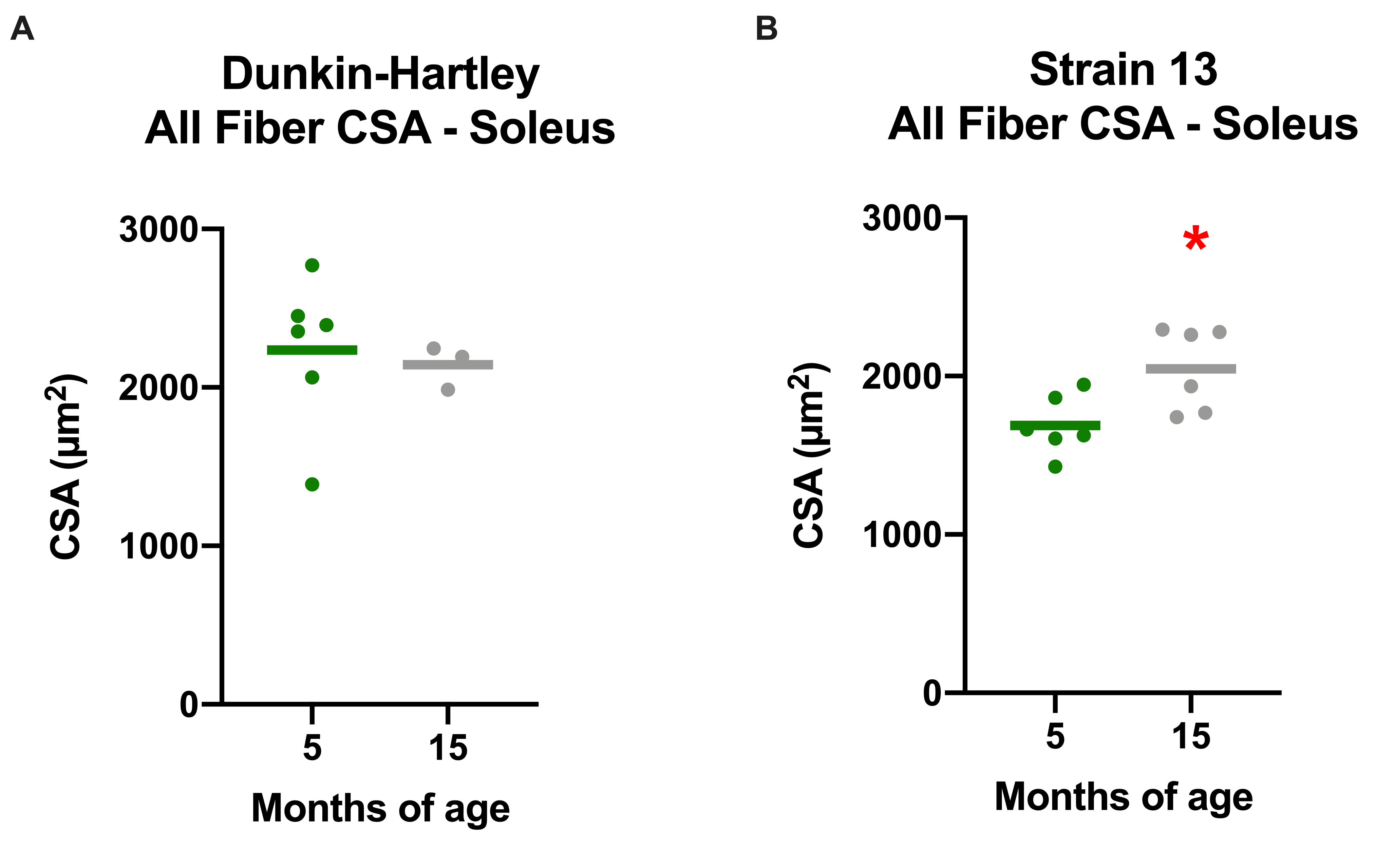
Supplementary Figures

**Supplementary Figure 1.** Cross sectional area of soleus myofibers of Dunkin Hartley and Strain 13 guinea pigs. There was no difference in average myofiber CSA of the soleus between 5 mo and 15 mo guinea pigs **(A)**. There was a significant difference (p<0.05) in myofiber CSA of the soleus between 5 mo and 15 mo Strain 13 guinea pigs **(B)**. n=3 for 15 mo Dunkin Hartley guinea pigs due to insufficient sample for cryosectioning for one guinea pig. * denotes p<0.05 compared to 5 mo.

**Supplementary Figure 2.** Muscle fiber type-specific CSA averages in the gastrocnemius of Strain 13 guinea pigs. There were no differences between the CSA of skeletal muscle fibers of any type in the gastrocnemii of 5 mo and 15 mo Strain 13 guinea pigs.


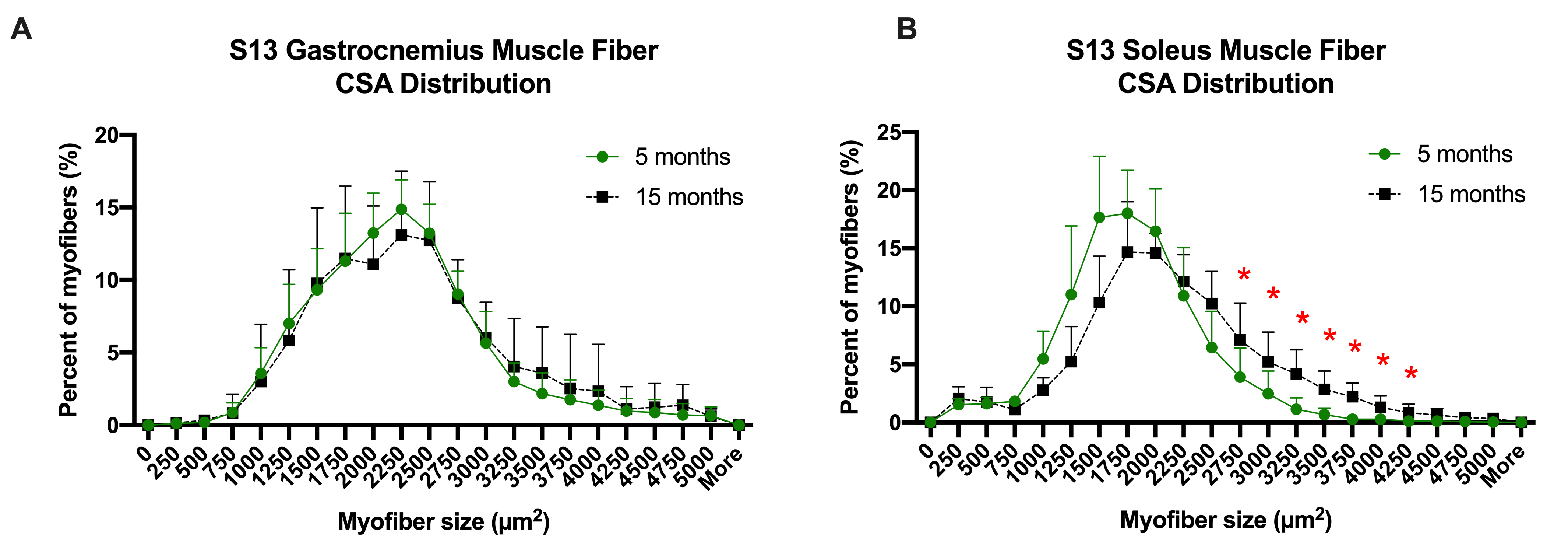


**Supplementary Figure 3.** CSA distribution of all myofibers in both the gastrocnemius and soleus of Strain 13 guinea pigs. There were no differences in myofiber size distribution in the gastrocnemius (A). But there were significant differences (p<0.05) in fiber size bins greater than 2750 µm^2^ in the soleus (B) of Strain 13 guinea pigs. * denotes p<0.05 compared to 5 mo.


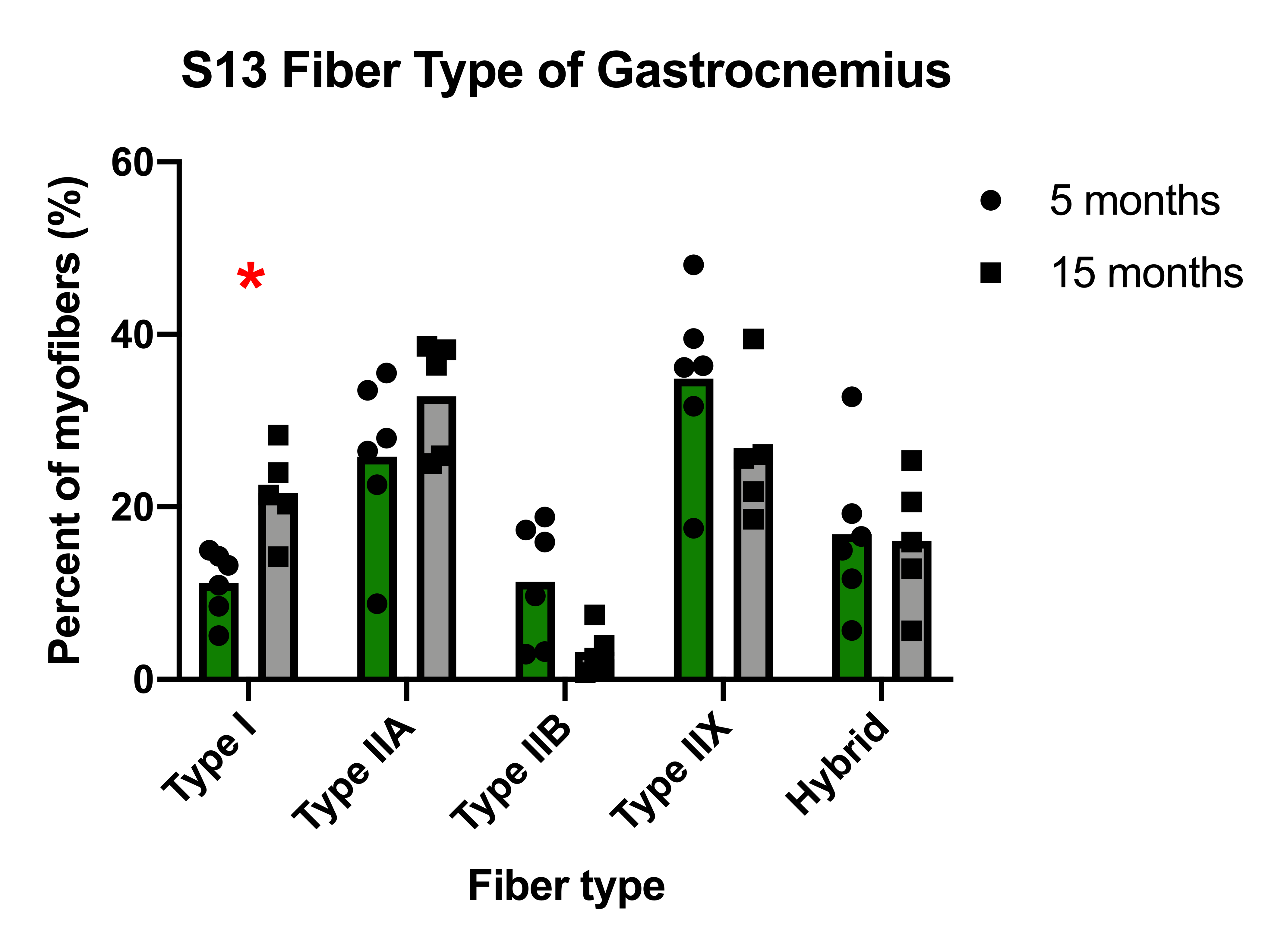


**Supplementary Figure 4.** Fiber type composition of the gastrocnemius of Strain 13 guinea pigs. There was a significant greater proportion of type I myofibers in 15 mo Strain 13 guinea pigs compared to 5 mo guinea pigs. There were no differences in the proportion of any type II myofibers.
